# Novel Peptide-Based PD1 Immunomodulators Demonstrate Efficacy in Infectious Disease Vaccines and Therapeutics

**DOI:** 10.3389/fimmu.2020.00264

**Published:** 2020-03-06

**Authors:** Vinayaka Kotraiah, Timothy W. Phares, Cecille D. Browne, James Pannucci, Marc Mansour, Amy R. Noe, Kenneth D. Tucker, Jayne M. Christen, Charles Reed, Alecia MacKay, Genevieve M. Weir, Rajkannan Rajagopalan, Marianne M. Stanford, Chun-Shiang Chung, Alfred Ayala, Jing Huang, Moriya Tsuji, Gabriel M. Gutierrez

**Affiliations:** ^1^Explorations in Global Health (ExGloH), Leidos Inc., Frederick, MD, United States; ^2^Leidos Life Sciences, Leidos Inc., Frederick, MD, United States; ^3^MM Scientific Consultants, Inc., Halifax, NS, Canada; ^4^Inovio Pharmaceuticals, Plymouth Meeting, PA, United States; ^5^IMV Inc., Dartmouth, NS, Canada; ^6^Lifespan-Rhode Island Hospital, Providence, RI, United States; ^7^The Aaron Diamond AIDS Research Center, New York, NY, United States

**Keywords:** vaccine, peptides, adjuvant, checkpoint, T-cell, cancer, sepsis, malaria

## Abstract

Many pathogens use the same immune evasion mechanisms as cancer cells. Patients with chronic infections have elevated levels of checkpoint receptors (e.g., programed cell death 1, PD1) on T cells. Monoclonal antibody (mAb)-based inhibitors to checkpoint receptors have also been shown to enhance T-cell responses in models of chronic infection. Therefore, inhibitors have the potential to act as a vaccine “adjuvant” by facilitating the expansion of vaccine antigen-specific T-cell repertoires. Here, we report the discovery and characterization of a peptide-based class of PD1 checkpoint inhibitors, which have a potent adaptive immunity adjuvant capability for vaccines against infectious diseases. Briefly, after identifying peptides that bind to the recombinant human PD1, we screened for *in vitro* efficacy in reporter assays and human peripheral blood mononuclear cells (PBMC) readouts. We first found the baseline *in vivo* performance of the peptides in a standard mouse oncology model that demonstrated equivalent efficacy compared to mAbs against the PD1 checkpoint. Subsequently, two strategies were used to demonstrate the utility of our peptides in infectious disease indications: (1) as a therapeutic in a bacteria-induced lethal sepsis model in which our peptides were found to increase survival with enhanced bacterial clearance and increased macrophage function; and (2) as an adjuvant in combination with a prophylactic malaria vaccine in which our peptides increased T-cell immunogenicity and the protective efficacy of the vaccine. Therefore, our peptides are promising as both a therapeutic agent and a vaccine adjuvant for infectious disease with a potentially safer and more cost-effective target product profile compared to mAbs. These findings are essential for deploying a new immunomodulatory regimen in infectious disease primary and clinical care settings.

## Introduction

Vaccinations have successfully provided protection from many life-threatening infectious diseases. Vaccines are also a cornerstone in disease eradication efforts. However, devastating infectious diseases that are not susceptible to classic vaccine approaches remain, despite decades of research and enormous public and commercial investments. All the vaccines currently in commercial and advanced developmental stages are designed to provide protection through the humoral pathway (1), despite other effector mechanisms, including CD8^+^ T cells that serve as the major protective immune mechanism against intracellular pathogens, such as *Leishmania* spp. and *Plasmodium* spp. (2), as well as viral infections, such as the hepatitis B virus, the human immunodeficiency virus, and influenza (3). Many of these pathogens have also evolved strategies to actively downregulate T-cell function by blocking naïve T-cell priming, and eventually exhausting T cells (2). Thus, overcoming these evasion strategies and boosting T-cell responses toward pathogen-derived vaccine antigens is a novel adjuvant strategy. The checkpoint receptors, such as programmed cell death 1 (PD1), represent a critical link in this pathogen-induced mechanism of immune evasion (4). Antagonizing the PD1 receptor (and other checkpoints) enables both the potentiation of the naïve-to-effector CD8^+^ T-cell transition and differentiation stage and restores CD8^+^ T-cell exhaustion in chronic infections. Therefore, PD1 inhibition embodies a critical target for use as a CD8^+^ T cell-inducing agent that can enhance prophylactic and therapeutic vaccines.

Although much attention has been focused on how checkpoint receptors and ligands are hijacked by cancer cells to avoid immune detection and elimination, the evidence that pathogens evade immunity via the same pathways is well-established, but not well-understood. Chronic viral and parasitic infections such as HIV, human T cell leukemia virus 1 (HTLV1), malaria, and helminths, are associated with T-cell exhaustion or extended hyporesponsiveness (2, 5–7). T cells become exhausted from continuous antigen exposure on the T-cell receptor (TCR) after having achieved effector function and then become inactive (8–15). Therefore, developing a truly effective therapeutic vaccine against these pathogens will also require reversing the negative signaling that causes the exhaustive state. An example is the HBV vaccine (Engerix-B), which is ineffective in chronically infected HBV patients (16, 17). *In vitro* studies of T cells isolated from chronically infected HBV patients have shown that the function can be partially restored by an antiPD1/PD-L1 blockade (18, 19). There is substantial evidence that targeting the checkpoint receptors improves disease state outcomes in animal models (15). For example, PD1 inhibition has been shown to reverse immune dysfunction and viral persistence in a mouse model of an HBV infection (12).

In a study by Bengsch et al. (20), the PD1 blockade of HBV inactive carrier patients' T cells (*ex vivo*) restored HBV-specific CD8^+^ T-cell function, which the authors linked to T-cell differentiation by increasing the naïve-to-effector CD8^+^ T-cell transition and differentiation. These findings agree with the observation that PD1 expression is highest in intermediate differentiated CD8^+^ T cells and with the published observation that exhausted HIV- and HCV-specific CD8^+^ T cells are linked to intermediate differentiation (21, 22). Moreover, in nonhuman primates, immunization with an adenovirus-based simian immunodeficiency virus (SIV) vaccine (SIVgag), in combination with an anti-PD1 monoclonal antibody (mAb), significantly increased peak SIV Gag-specific T-cell responses, suggesting there is an adjuvant-like prophylactic effect when PD1 is blocked (23). Therefore, antagonizing PD1 in a prophylactic or therapeutic vaccination would enable greater expansion of antigen-specific T cells, resulting in amplified recall responses and reversed exhaustion.

The anti-PD1 mAb tools developed to treat cancer do not readily lend themselves to the field of infectious disease vaccinology. Monoclonal antibodies are large molecules that, while effective, can produce severe immune-related events and precipitate autoimmune disease (e.g., type 1 diabetes) because of their long serum half-life that results in an extended activation of the immune pathway (24, 25). Thus, administering mAbs to a healthy or asymptomatic population as a prophylactic vaccine adjuvant presents an unacceptable safety risk. mAbs would also be extremely difficult to formulate and deliver with an infectious disease vaccine in the field. The high economic cost of mAbs is a significant hurdle for the most at-risk populations. Thus, PD1 antagonists based on small molecules such as a peptide, represent a favorable alternative because these are compatible with different routes of administration and different formulations, and have a significantly lower cost than mAbs. A peptide-based inhibitor's shorter half-life will also provide greater control over bioavailability to potentially offer a significantly reduced safety risk. Therefore, peptides represent an ideal modality as a checkpoint inhibitor-based adjuvant for infectious disease vaccines.

Here, we report the identification of novel peptide-based PD1 immunomodulators that can be deployed for cancer and as T-cell adjuvants for vaccines targeting infectious disease. Screening random peptide phage libraries revealed a series of small linear peptides, namely PD1 peptides, which are bound to the human PD1 receptor and were shown to competitively inhibit PD-L1 binding in *in vitro* assays. *In silico* docking models demonstrate that our PD1 peptides potentially bind to unique domains of the receptor. *In vivo*, the anti-PD1 peptides block metastases similarly to anti-PD1 mAb in the lungs of B16 melanoma mouse models. These studies became the basis for us to further apply our peptides as therapeutics in a lethal mouse model of sepsis. Sepsis-induced animals treated with our peptides had an increased survival rate that correlated with a decreased bacterial load and an alleviation of macrophage dysfunction. Finally, to demonstrate their vaccine adjuvant capacity, we combined our peptides with a prophylactic malaria vaccine in an animal model. PD1 peptide-treated mice that received an experimental malaria vaccine showed an increase in protection against malaria, which correlated with an increased level of malaria antigen-specific CD8^+^ T-cell response. Thus, we demonstrate the utility of anti-PD1 peptides as potential therapeutics for cancer and sepsis as well as their promise as T-cell adjuvants for infectious disease vaccines.

## Materials and Methods

### Phage Display

An M13-based phage library was prepared by pooling three peptide libraries: TriCo-20 and TriCo-16 (Creative Biolabs, Shirley, NY, USA) and PhD-12 (New England Biolabs, Ipswich, MA, USA). Soluble recombinant PD1 (Creative Biolabs) were coated in 96-well plates at 1.5 μg in 50μL of coating buffer (0.1 M NaHCO_3_, pH 8.6), and the plates were incubated at 4°C overnight. After shaking out the coating solution, the wells were blocked with 3% PBSM (PBS [phosphate buffered saline] containing 3% skim milk) at 37°C for 2 h. In each panning round, the plates were incubated with the pooled phage library for 2 h at 37°C at an input titer of 10^9^ to 10^11^. After washing ten times with Tris-buffered saline (TBS) or PBS containing Tween-20 (0.1% Tween-20 in the first four panning rounds and 0.2% Tween-20 in the fifth panning round), bound phage was eluted with glycine-HCl, pH 2.2. Each round consisted of incubations in PD1-coated plates and plates without PD1 (for non-specifically bound phages) and subsequent elution of phages bound to PD1. Eluted phages were then amplified using *Escherichia coli* ER2738 and tested by phage ELISA. For phage ELISA, PD1 was coated at 20 μg/mL in a 96-well plate and incubated with phage (amplified polyclonal eluate or individual clones). After washing, bound phage was detected by mouse anti-M13 antibody conjugated to HRP (GE Healthcare Life Sciences, Marlborough, MA, USA). Colorimetric signals were measured by absorbance at 450 nm. Signals from PD1-coated plates were divided by signals from wells that were not coated with PD1 to determine normalized signals.

### Peptide Synthesis

Following four and five rounds of biopanning, phage clones were selected for sequencing. The inserted DNA (encoding the foreign peptide) was amplified by a polymerase chain reaction. Amplified DNA fragments from individual clones were sequenced by Creative Biogene (Shirley, NY, USA). Peptide sequences corresponding to the DNA sequences were analyzed using the *ClustalX* software to align peptide sequences and a NCBI BLAST search to identify proteins with motifs that were homologous to the peptide sequences. All peptides were synthesized by the standard Fmoc method using a peptide synthesizer and purified by high-performance liquid chromatography to >90% purity, and peptide mass was confirmed by matrix-assisted laser desorption ionization-time of flight (Creative Peptides, Shirley, NY, USA).

### Cell-Binding Assay and Competitive Inhibition

The Jurkat T-cell line that was used in competitive inhibition was a recombinant Jurkat T-cell line that was purchased from Promega (Madison, WI, USA; Cat. 60535) and was cultured according to the manufacturer's instructions. This cell line expressed the firefly luciferase gene under the control of the nuclear factor of activated T cells (NFAT) response elements and overexpressed human PD-1 (Programmed Cell Death 1, GenBank Accession #NM_005018). PD1 protein expression was detected using anti-human PD1 conjugated to allophycocyanin (APC) (BioLegend, San Diego, CA, USA). Jurkat cells were added to wells of a 96-well plate. An 11-point dilution series of each peptide starting at 10 μM was prepared. Peptide dilutions were added to wells containing 50,000 Jurkat cells per well and incubated for 30 min on ice. Following incubation, cells were washed with staining buffer, and then incubated with recombinant human PDL1-Fc fusion protein (Life Technologies, Carlsbad, CA) at 4 μg/mL for 30 min on ice. Cells were then washed with staining buffer and incubated with anti-human IgG conjugated to AF647 (Jackson ImmunoResearch, West Grove, PA, USA) for 30 min on ice. Cells were washed and re-suspended with staining buffer and analyzed by flow cytometry.

### Cell-Based Reporter Assay

The PD1/PD-L1 blockade assay kit was purchased from Promega (Catalog #CS187106). The assay kit included a reagent for the detection of luminescence (Bio-Glo) as well as two cells lines: an APC called aAPC/CHO-K1 and a Jurkat T-cell line. The Jurkat T-cell line is the same cell line used in the competitive inhibition assay. The aAPC/CHO-K1-cell line is a Chinese hamster ovary cell line that expresses human PD-L1. The assay was performed according to the manufacturer's instructions. Briefly, aAPC/CHO-K1 cells were plated and incubated overnight in a 37°C CO_2_ incubator. Dilutions of peptide inhibitors were added to wells and incubated at ambient temperature while preparing the effectors cells (Jurkat). Jurkat cells were added to wells, and the plate was incubated at 37°C CO_2_ incubator for 6 h. Bio-Glo Reagent was added to wells and the plate was incubated at ambient temperature for 5–30 min. Luminescence signals were measured using a PerkinElmer EnVision Xcite Multilabel plate reader (PerkinElmer Life and Analytical Sciences, Shelton, CT, USA). Fold induction was calculated by: Fold induction = Relative Light Units - RLU (induced minus background)/RLU (no antibody control minus background).

### Biacore Analysis

Binding experiments were performed on a Biacore T-200 (GE Healthcare Life Sciences, Buckinghamshire, UK) at 25°C. The assay buffer was 10 mM HEPES buffer (pH 7.4), 150 mM NaCl, 3 mM EDTA, and 0.05% P20. The regeneration buffer was 10 mM HEPES buffer (pH 7.4), 150 mM NaCl, 3 mM EDTA, and 0.05% P20. The immobilization buffer was 10 mM sodium acetate at pH 5.0. The flow rate used for immobilizing the ligand was 5 μL/min. The flow rate for kinetics analysis was 30 μL/min. In initial scouting experiments, 12,000 RU of human and 6000 RU of mouse PD1 receptors were directly immobilized on flow cell 2 and flow cell 4 of the CM5 chip using an amine coupling method (EDC/NHS). The unoccupied sites were blocked with 1M ethanol amine. Scouting was performed at a single analyte (human or mouse PD-L1) concentration of 25 μM to confirm binding. Flow cell 1 was kept blank and used for reference subtraction. Binding of an analyte to the ligand was monitored in real-time. Based on the scouting results, full kinetics were performed by immobilizing 10,000 RU of the ligand to a new chip using an analyte concentration of 25 μM, followed by serial dilution to 12.5, 6.25, 3.125, 1.562, 0.78, and 0 μM concentration, or as indicated. Data analysis was performed using software for Biacore 3000, because it has more flexibility in terms of obtaining the binding characteristics with a limited analyte concentration range. Because of the fast on and off rates, equilibrium dissociation constant (KD) was determined using steady-state equilibrium kinetics. Chi-square (χ^2^) analysis was performed between the actual sensorgram and the sensorgram generated from the BIAnalysis software to determine the accuracy of the analysis. A χ^2^ value within 1–2 was considered to be significant (accurate) and below 1 was considered to be highly significant (highly accurate).

### Tetanus Toxoid Recall Assay

A previously screened human PBMC sample, A3327, was thawed. Sample A3327 was obtained from a human donor through an informed consent protocol (Informed Consent in White Blood Cell Collection) approved by the Western Institutional Review Board (Puyallup, Washington). Cells were counted and the viability was verified. Then, 800,000 PBMCs in 100 μL were added to a 96-well round bottom plate. A tetanus toxin was added into a 50 μL volume to a final concentration of 5 μg/mL (1 μg total). The test peptides were added in a volume of 50 μL at concentrations ranging from 25 to 200 μM. Treated cells were cultured at 37°C for 4 days. After incubation, 150 μL of supernatant was harvested for subsequent ELISA analysis of cytokines. Seven different cytokines were measured (interleukin [IL]−6, IL-17A, interferon [IFN]-γ, and tumor necrosis factor [TNF]-α).

### PD1 Peptide Docking and Analysis

Peptide sequences were subjected to molecular docking to the extracellular portion of the human PD-1 molecule. The specific sequence was identified and a reliable model was built based on the known crystal structures. CABS-dock (26, 27), a peptide docking algorithm that features flexible peptide searches was used for docking peptides for the ligand. Top positions from the CABS-dock were superimposed and inspected, and atomic positions were refined and minimized. Results correlated in general with the docking cluster ranks.

### Peptide Formulation

Individual peptides were reconstituted at 50 mg/mL in 0.25% acetic acid. They were then diluted in a specific order (QP20, HD20, WQ20, and SQ20 from first to last) into a 0.1M sodium acetate solution (pH adjusted to 9.5 with 0.5M NaOH) so that the final concentration of each peptide was 2 mg/mL (8 mg/mL in total). The pH of this combination solution was around 5.5, and this formulation was injected into mice.

### B16-F10 Melanoma Model

Briefly, on study day 0, mice (*n* = 5–6) were implanted with B16-F10-LacZ tumor cells (2.5 × 10^5^, 5 × 10^5^, or 1 × 10^6^) by intravenous injection into the tail vein. Mice received 200 μg/injection/mouse of PD1 peptide antagonists or anti-PD1 mAb administered on days 1, 2, 4, 6, 8, and 12 or days 2, 5, 7, 9, and 12 (data not shown) after injection of tumor cells. Detailed clinical examinations and body weights were recorded after each treatment. Mice were sacrificed on study day 14, their lungs removed, stained, and the number of tumor metastases were counted.

### Animals and Sepsis Model of Cecal Ligation and Puncture

Male C57BL/6 mice were purchased from Jackson Laboratories (Bar Harbor, ME, USA). The mice (8–10 weeks old) were housed in a room with an ambient temperature of 22°C and a 12:12-h light-dark cycle and allowed to acclimatize in the animal facility for 1 week before using them in the experiments. Experiments were conducted rigorously with appropriate controls and replication with sample sizes large enough to produce robust results and conducted in accordance with National Institutes of Health guidelines and with approval from the Animal Use Committee of Rhode Island Hospital (#5025-17).

Polymicrobial sepsis was induced in mice using the cecal ligation and puncture (CLP) model (28). Mice were anesthetized with isofluorane, their abdomen shaved and scrubbed three times with betadine-alcohol. A ventral midline incision (1.0–1.5 cm) was made below the diaphragm to expose the cecum. The cecum was ligated with 5-0 silk, punctured twice with a 21-gauge needle and gently compressed to extrude a small amount of fecal contents through the punctured holes. The cecum was returned to the abdomen and the incision was closed in layers with 6-0 Ethilon suture (Ethicon, Inc., Somerville, NJ, USA). All animals were then resuscitated with 0.6 mL of lactated Ringer's solution SC. At 3 h and 9 h post-CLP, the vehicle buffer or peptide solutions (200 μg/100 μL buffer) were injected intra-peritoneally (IP). Anti-PD1 antibody (RMP-1-14, Bio X Cell; 200 μg/100 μL PBS) were injected IP once at 3 h post-CLP. For survival studies, mice received two injections/day of vehicle or PD1 peptides for 3 days or one injection of anti-PD-1 antibody/day for 5 days post-CLP, and their survival was monitored for 7 days.

All procedures, including surgical procedures, monitoring and endpoints, were approved by IACUC, including the use of local analgesics as systemic analgesics are contraindicated for our work and the withholding of which was approved by the IACUC. All efforts were made to minimize the number of animals used and their suffering. For studies looking at absolute survival, alternatives were considered, which included surrogate endpoints, such as decreased body temperature, clinical condition, decreased body weight and inability to ambulate. Explicitly, these survival studies, including death as a potential endpoint, were approved, including the percentage of mortality anticipated for this work and the inability to use surrogate end points for this specific experiment while still generating a meaningful set of replicates. However, these specific humane endpoints mentioned were adopted for all other studies involving our mouse model of sepsis included in this manuscript; thus, animals were either humanely euthanized at their experimental endpoint or when a surrogate humane endpoint was reached.

### Sample Preparation for Blood and Peritoneal Fluids in CLP-Induced Sepsis

Twenty-four h post-procedure, mice were euthanized by CO_2_ asphyxiation. Blood was collected by cardiac puncture for bacterial burden. Peritoneal fluids and cells were obtained from mice by lavage of the peritoneal cavity. For the bacterial burden, lavage peritoneal fluids were collected after injecting 1 mL of PBS into the peritoneum, clarified by centrifugation (800 *g* at 4°C for 15 min) and used for the bacterial count by serially diluting in PBS and plating on blood agar plates.

### Preparation of Peritoneal Macrophages for Phagocytosis Assay

Peritoneal macrophages were obtained from mice through a peritoneal cavity lavage. Cells were collected after injecting 1 mL of PBS into the peritoneum and centrifugation (800 g at 4°C for 15 min). A second lavage was performed using 4 mL of PBS to collect more cells. The cells were combined from the two lavage collections, resuspended in DMEM with 10% FBS at 1 × 10^6^ cells/mL and plated onto plastic tissue culture plates (12-well), followed by incubation at 37°C for 6–12 h. After incubation, non-adherent cells were removed by washing twice with fresh DMEM. Adherent macrophages were then co-cultured with pHrodo BioParticles-conjugated *E. coli* (Molecular Probes, Eugene, OR, USA) at 37°C for 1 h and washed completely with PBS (29). Cells were scraped from the plates, stained with anti-F4/80 for 30 min, washed, and analyzed by flow cytometry. Macrophage phagocytic efficiency was evaluated by MACSQuant Analyzer (Miltenyi, Auburn, CA, USA). Peritoneal macrophages were labeled with pHrodo BioParticles (red) and APC-anti-F4/80 for phagocytosis analysis. Data were analyzed with the FlowJo software (29–31).

### Bacterial Burden

Blood and peritoneal lavage fluids (in PBS) were plated on trypticase soy agar with 5% sheep blood plates (BD Bioscience, San Jose, CA, USA), cultured at 37°C for 24 h and colonies on the plates were counted (29).

### Mice, Malaria Vaccine, and Parasites

Six- to eight-week-old female BALB/c mice were purchased from Taconic (Germantown, NY, USA). All mice were maintained under standard conditions in the Laboratory Animal Research Center of the Rockefeller University, and the protocol was approved by the Institutional Animal Care and Use Committee at the Rockefeller University (Assurance no. A3081-01). A recombinant serotype 5 adenovirus, AdPyCS that expressed *Plasmodium yoelii* circumsporozoite protein (PyCS) was constructed as previously described (32). Wild-type *P. yoelii* parasites of the 17 XNL strain were maintained in the insect facility of the Division of Parasitology, Department of Microbiology at New York University School of Medicine. *P. yoelii* sporozoites were obtained from dissected salivary glands of infected *Anopheles stephensi* mosquitoes 2 weeks after an infective blood meal (32, 33).

### Assessment of the Level of Antigen-Specific CD8^+^ T-Cell Response by an ELISpot Assay

The relative numbers of PyCS-specific, IFN-γ-secreting CD8^+^ T cells among whole splenocytes isolated from the spleens of immunized mice were determined by an ELISpot assay, using a mouse IFN-γ ELISpot kit (Abcam, Cambridge, MA, USA) and a synthetic 9-mer peptide, SYVPSAEQI (purchased from Biosynthesis Inc.; Lewisville, TX, USA) corresponding to the immunodominant CD8^+^ T-cell epitope within the PyCS antigen, as previously described (32–34) with some modifications. Briefly, splenocytes were prepared by lysing red blood cells from a single cell suspension obtained from a spleen that was collected from mice 12 days after immunization, and 5 × 10^5^ splenocytes/well were incubated with 5 μg/mL of the peptide for 24 h at 37°C on the ELISpot plate pre-coated with an IFN-γ antibody, as previously described (32–34). Subsequently, the ELISpot plate was incubated with biotinylated anti-mouse IFN-γ antibody, followed by incubation with avidin-conjugated with horseradish peroxidase. Finally, the spots were developed after adding ELISpot substrate (Abcam). To identify the number of IFN-γ-secreting CD8^+^ T cells in each well, the mean number of spots (for duplicates) counted in the wells incubated with splenocytes in the presence of the peptide was subtracted by the mean number of spots (for duplicates) counted in the wells that were incubated with splenocytes only.

### Assessment of the Level of Antigen-Specific CD8^+^ T-Cell Response by Tetramer

The percentage of PyCS-specific CD8^+^ T cells among splenocytes of immunized mice were determined by a tetramer assay, as we previously described (34). Briefly, after isolating splenocytes as described above, the cells were washed twice and blocked for 5 min on ice using inactivated normal mouse serum supplemented with anti-CD16/CD32 (clone 93 – BioLegend, San Diego, CA, USA). Then, cells were stained for 40 min on ice in the dark with the following antibodies: anti-mouse CD3 (clone SK7, BioLegend), anti-mouse CD8 (clone SK1, BioLegend), and H-2K^d^/SYVPSAEQI-tetramer (provided by an NIH tetramer core facility). After staining, cells were washed twice with PBS containing 2% FBS, fixed with 1% paraformaldehyde, and the staining profiles were acquired using a BD LSR II (BD Biosciences, Franklin Lakes, NJ, USA), using FACS DIVA software. Data analyses were performed using FlowJo Software version 10.0.6 (Tree Star Inc., Ashland, OR, USA) (34).

### Sporozoite Challenge and Assessment of Protection

Sporozoite challenge experiments were performed as described previously (32–35). Briefly, immunized mice were administered 100 live *P. yoelii* sporozoites IV via a tail vein. Parasitemia was monitored from days 3 to 8 after the sporozoite challenge, by detecting the presence of parasitized red blood cells in thin blood smears to assess for a complete protection against malaria. Briefly, a drop of blood was collected from the mouse tail vein for thin blood smears on pre-cleaned glass slides. Thin blood smears were fixed with absolute methanol and then stained with diluted Giemsa stain (1:20, v/v) (Sigma-Aldrich, St. Louis, MO, USA) for 20 min. The presence of parasitemia (parasitized red blood cells) was examined using a 100 × oil immersion objective under the microscope.

### Statistical Analysis

All the statistical analyses were performed using GraphPad Prism (GraphPad Software, Inc., La Jolla, CA, USA). Bars in each figure represent mean ± standard deviation (SD). One-way analysis of variance (ANOVA) followed by a Dunnett's test was used to determine the differences between three or more groups, whereas, an unpaired *t*-test was used if the comparison was performed between two groups.

## Results

### Identification of PD1-Binding Peptides by Phage Display

**Figure 1:**
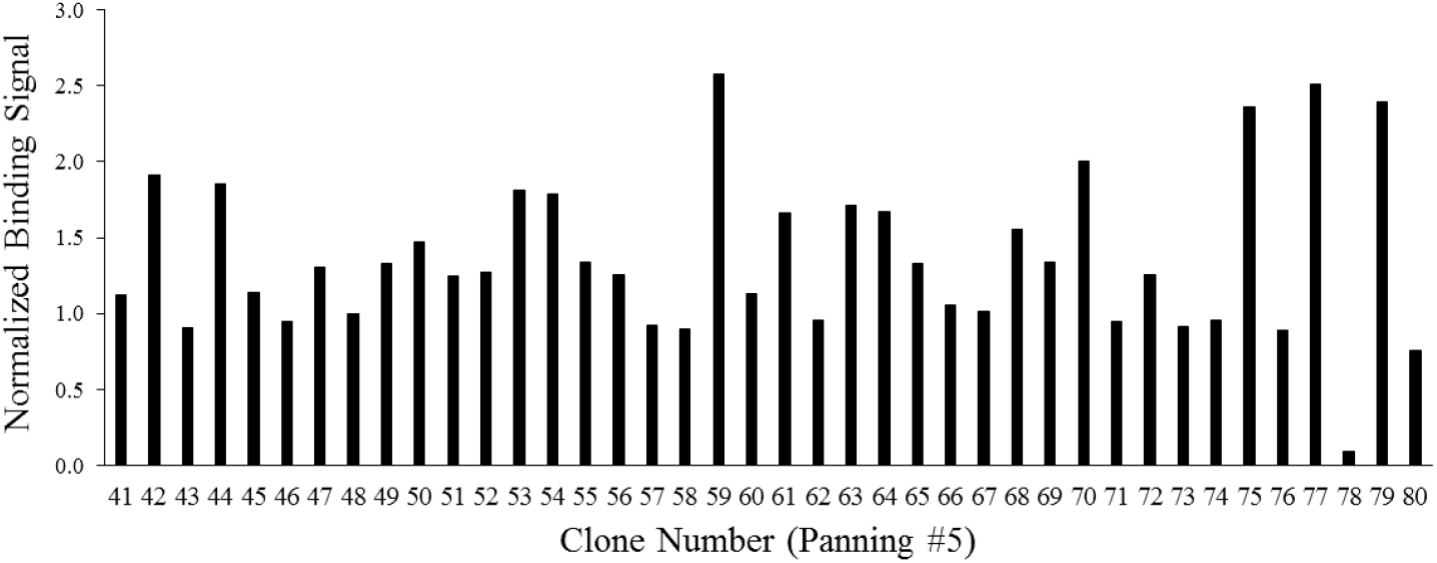
Phage ELISA signals of 40 isolated clones after the fifth panning. PD1-coated wells of a 96-well plate were incubated with amplified phage clones, and phage binding was detected with a monoclonal antibody to M13. Colorimetric signals (absorbance at 450 nm) were normalized against signals from wells with no PD1 incubated with the corresponding phage clone. Signals are averages of duplicate wells.

A peptide phage display technique based on pooled phage libraries was used to identify peptides that bind human PD1. Screening was performed in a succession of five phage panning steps. In each panning step, PD1 was coated on plates and incubated with the phage library. Unbound phages were removed by washing, and then bound phages were eluted, amplified, and analyzed by phage ELISA. The panning process was repeated four more times with increasing washing stringency, and after each panning, the degree of enrichment based on phage ELISA was monitored (Figure S1). After the fifth panning, clones were randomly isolated and analyzed by phage ELISA. The normalized binding signals of selected clones are shown in Figure 1. DNA sequencing of the selected clones revealed the peptide sequence displayed by each phage clone. Four clones were selected for peptide synthesis, labeled as QP20 (Clone #42), HD20 (Clone #59), WQ20 (Clone #79), and SQ20 (Clone #54). The clone identification (ID) number, frequency, and peptide ID number of selected peptides are listed in Table 1.

**Table 1:** Selected peptides.

**Clone ID**	**Frequency**	**Peptide ID**
42	4	QP20
59	2	HD20
79	1	WQ20
54	2	SQ20

### Binding of Peptides to Human and Mouse PD1

The four peptides identified by phage display were then tested for binding to human and mouse PD1 using the surface plasmon resonance technique. After initial scouting results, a full kinetics analysis was performed by immobilizing the peptide and analyzing the serially diluted receptor protein at seven different concentrations. Because of the fast on and off rates, KD was determined by steady-state equilibrium kinetics. The KD values are shown in Table 2. The data indicates that all four peptides could bind both human and mouse PD1.

**Table 2:** Binding of peptides to human and mouse PD1 protein.

**Ligand**	**Analyte**	**Rmax (RU)**	**KA (1/M)**	**KD (M)**	**Conc. (μM)**	**Chi^**2**^**
Mouse PD1	WQ-20	270	1.31 × 10^3^	7.61 × 10^−4^	0–25	0.0203
Mouse PD1	QP-20	13.4	1.80 × 10^4^	5.54 × 10^−5^	0–25	0.0446
Mouse PD1	HD-20	76	4.25 × 10^3^	2.35 × 10^−4^	0–25	0.11
Mouse PD1	SQ-20	12.8	2.14 × 10^4^	4.68 × 10^−5^	0–25	0.039
Human PD1	WQ-20	84.7	3.28 × 10^3^	3.05 × 10^−4^	0–25	0.0309
Human PD1	QP-20	3.83	9.36 × 10^4^	1.07 × 10^−5^	0–25	0.0569
Human PD1	HD-20	3.35	3.18 × 10^5^	3.41 × 10^−6^	0–12.5	0.0733
Human PD1	SQ-20	4.05	1.94 × 10^5^	5.16 × 10^−6^	0–25	0.111
Mouse PD1	Mouse PD-L1	1.07 × 10^3^	6.50 × 10^5^	1.64 × 10^−6^	0–1000	2.27
Human PD1	Human PD-L1	2.31 × 10^3^	3.98 × 10^5^	2.51 × 10^−6^	0–1000	4.44
Mouse PD1	Mouse PD-L1	259	2.75 × 10^6^	3.64 × 10^−7^	0–50	0.105
Human PD1	Human PD-L1	213	6.92 × 10^6^	1.44 × 10^−7^	0–50	2.44

*RU is Response Units; Rmax is maximum binding capacity (in RU) of ligand captured/immobilized on the surface; KA is the association rate constant; KD is the equilibrium binding affinity constant; Conc is the concentration range of the analyte; Chi^2^ value was calculated on the observed and the fitted curve to determine the accuracy of the fitted curve compared to the measured curve*.

### PD1-Binding Peptides Bind to Jurkat Cells Overexpressing PD1

**Figure 2:**
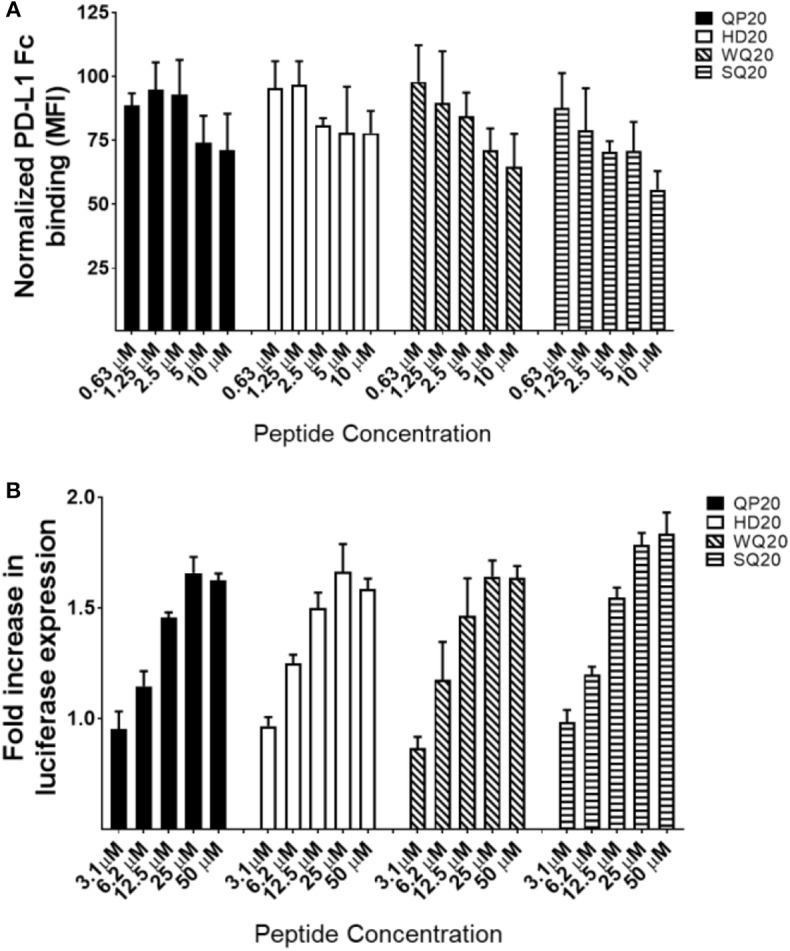
Binding of peptides to PD1 on Jurkat T cells. **(A)** Jurkat T cells (effector cells) were pre-incubated with varying amounts of each peptide and then cells were washed and incubated with PD-L1-Fc protein. Bound PD-L1-Fc was detected with anti-human IgG conjugated to AF647, and cells were analyzed by flow cytometry. MFIs were normalized (per sample) against samples without peptide. Duplicate samples were tested. Error bars represent the standards of deviation. **(B)** Interference with the interaction between effector T cells (Jurkat) and target cells (CHO); target cells were cultured overnight and then incubated Jurkat cells with varying amounts of peptides in triplicate cells. After 6 h, luminescence signals were measured. Fold increase in luminescence was calculated by dividing the RLU (relative luminescence units) of the treated cells by the RLU of the untreated cells.

To determine if the PD1-binding peptides identified by phage display also bind PD1 displayed on a cell surface, a Jurkat T cell line that overexpresses human PD1 was used. PD1 expression on these engineered Jurkat cells was verified by flow cytometry using anti-human PD1 conjugated to allophycocyanin (APC) (Figure S2). PD-L1-Fc binding to these Jurkat cells was also verified by detection with anti-human IgG conjugated to AF647 and by measurement of the mean fluorescence intensities (MFI) by flow cytometry (Figure S3). To determine if QP20, HD20, WQ20, and SQ20 bind the human PD1 on the cell surface, peptides were serially diluted and incubated with Jurkat cells. Cells were subsequently washed with a buffer and incubated with PD-L1-Fc. Bound PD-L1-Fc was then detected with anti-human IgG conjugated to AF647. Results show that the pre-incubation of cells with increasing amounts of peptides leads to a reduction in PD-L1-Fc binding. At 10 μM, peptides QP20 and HD20 showed reductions in PD-L1-Fc binding of <30%, while WQ20 and SQ20 showed reductions in PD-L1-Fc binding of >30% (Figure 2A). These results indicate that the peptides bind to PD1 on the cell surface and suggest that these peptides have the ability to block the binding of PD-L1 to PD1.

### PD1-Binding Peptides Inhibit PD1 Receptor Signaling

To determine the ability of the PD1-binding peptides to interfere with the engagement of PD1 to PD-L1, a cell-based reporter assay was performed in which the intercellular interaction between PD1-expressing Jurkat T effector cells and PD-L1-expressing Chinese hamster ovary cells (CHO) was evaluated in the presence of PD1-binding peptides. The PD1/PD-L1 blockade cell-based assay is a bioluminescent cell-based assay that measures the interaction between Jurkat T cells overexpressing human PD-1, as well as a luciferase reporter driven by an NFAT response element (NFAT-RE) and APC/CHO-K1 cells expressing human PD-L1. When the two cell types are co-cultured, the PD-1/PD-L1 interaction inhibits TCR signaling resulting in inhibition of NFAT-RE-mediated luminescence. The addition of an anti-PD1 inhibitory antibody blocks the PD-1/PD-L1 interaction and releases the inhibitory signal resulting in TCR activation and NFAT-RE-mediated bioluminescence (Figure S4). To determine if HD20, QP20, SQ20, and WQ20 peptides inhibit the interaction between PD1 and PD-L1 in this co-culture system, peptides were added to the co-culture at varying amounts, and the levels of NFAT-RE-mediated bioluminescence were measured. Results show that all four peptides induced bioluminescence (induction of TCR signaling) in a dose-dependent manner, and a peak fold increase was seen at >1.5 with 25 μM (Figure 2B). These results indicate that the peptides prevent the natively displayed PD-L1 on target cells from interacting with the natively displayed PD1 on effector cells resulting in enhanced T cell activation.

### PD1 Peptides Enhance Antigen-Specific Cell-Mediated Responses *in vitro*

**Figure 3:**
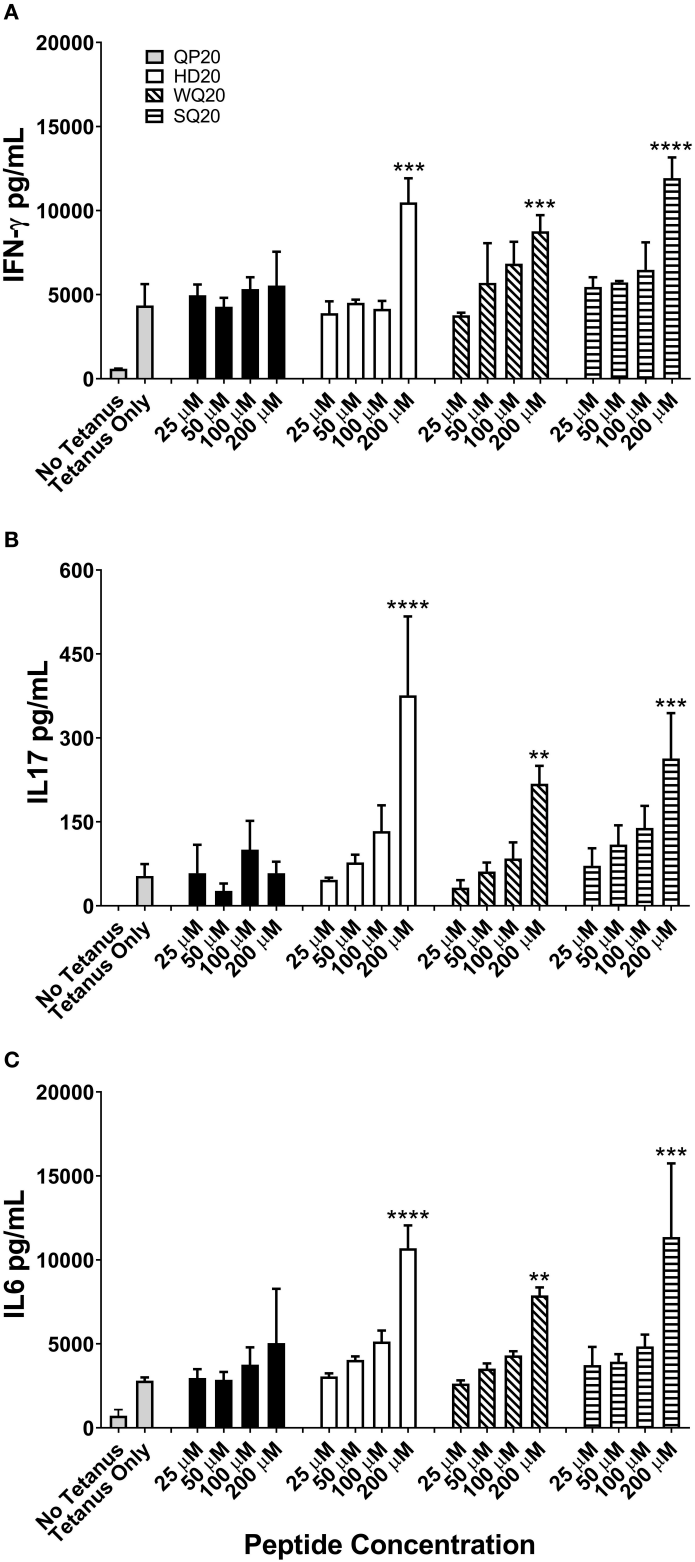
PD1-binding peptides enhance cytokine secretion from tetanus toxoid stimulated human PBMCs. **(A–C)** PBMCs from a single donor were stimulated with tetanus antigen (5 μg/mL). Individual PD1-binding peptides were added at concentrations ranging from 25 to 200 μM. Cells were cultured for 4 days and supernatant IFN-γ **(A)**, IL-17 **(B)**, and IL-6 **(C)** levels were measured by ELISA. Data are expressed as the mean ± standard deviation (SD). Significant differences between tetanus only and PD1-binding peptide treated PBMCs were determined using a one-way ANOVA multiple comparisons test, and significance is denoted by ^**^p < 0.01, ^***^p < 0.001, and ^****^p < 0.0005.

A human PBMC-based tetanus antigen recall assay was used to test whether PD1 inhibitor peptides can modulate T-cell responses measured by cytokine production. PBMCs from a pre-screened human donor with measurable tetanus antibody titers were stimulated with 5 μg/mL of a tetanus antigen and incubated with various doses of each PD1-binding peptide for 4 days. After incubation, supernatants were harvested for subsequent ELISA analysis of IFN-γ, IL-17, and IL-6 (Figure 3). With the exception of QP20, the other PD1-binding peptides significantly increased cytokine secretion at 200 μM relative to tetanus alone, which supports the hypothesis that the PD1-binding peptides enhance antigen-specific T-cell responses.

### Molecular Docking of PD1 Binding Peptides to PD1

**Figure 4:**
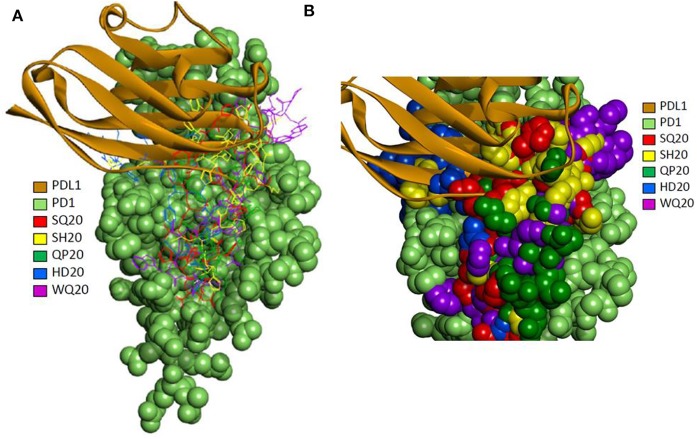
**(A)** Molecular docking of PD1 binding peptides to PD1. Experimentally determined binding pose for human PD-L1 is shown superimposed with the top predicted poses for peptides SQ20, QP20, HD20, and WQ20 docked on human PD1. **(B)** Zoomed-in view of predicted poses for peptides. Note that SH20 (clone 75 in Figure 1) is another peptide that was also isolated as a PD1 binder in the phage library screen and is shown here for comparison to the four peptides.

A set of peptides was predicted to overlap with the known PD-L1 interacting site based on the 4 zqk crystal structure (36). The selected top poses for each of the five peptides were analyzed for potential molecular interactions on the PD1 surface. The predicted peptide locations and PD-L1 occupied locations on the receptor that were only partially overlapping (Figure 4A). The models suggest that different peptides occupy somewhat exclusive locations on the receptor (Figure 4B).

### Systemic Therapy With PD1 Peptide Reduces B16-F10 Lung Metastases

Based on the large number of studies assessing the impact of checkpoint inhibitors in cancer and the use of anti-PD1 antibodies as a cancer treatment, we selected a cancer model to conduct the initial assessments of the PD1 peptide antagonist efficacy *in vivo*. The B16-F10 mouse melanoma model was selected because this model is sensitive to anti-PD1 treatment. In an initial study, SQ20 and QP20 peptides were selected for testing because they are bound to mouse PD1 with a higher affinity compared to WQ20 and HD20 (Table 2). Additionally, the combination of all four peptides and the anti-PD1 mAb were also tested to evaluate the potential for synergy among the PD1 peptide antagonists. The group that received the four-peptide combination showed the fewest surface tumor metastases compared to the groups receiving anti-PD1 mAb (positive control) or the two individual peptides (data not shown). Therefore, subsequent studies on this model were focused on assessing the four peptides in combination.

**Figure 5:**
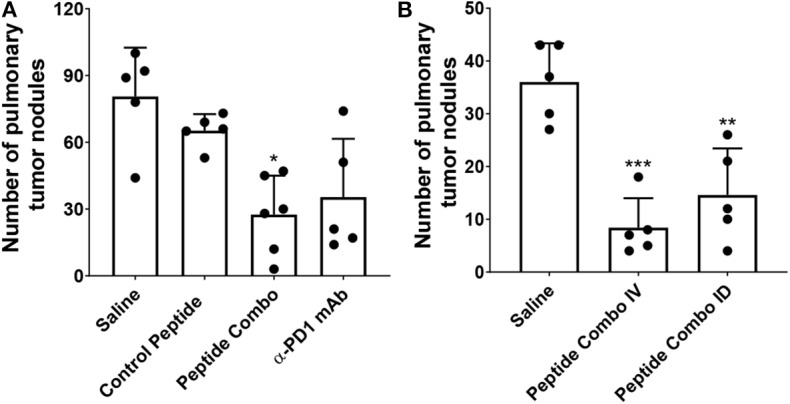
Decreased tumor metastasis following treatment with PD1 peptide antagonists in a syngeneic model of B16-F10 mouse melanoma. **(A)** Mice (*n* = 5–6) were injected IV with LacZ expressing B16 melanoma cells and treated IV with control peptide, PD1 peptide antagonists, or α-PD1 mAb. Control peptide is the myelin oligodendrocyte glycoprotein_35−55_. The pulmonary tumor nodules were counted on day 14. **(B)** Mice (*n* = 5) were injected IV with LacZ-expressing B16 melanoma cells and treated either IV or ID with PD1 peptide antagonists. The pulmonary tumor nodules were counted at day 14. Data are expressed as the mean ± SD. Significant differences between control peptide and treated mice in **(A)** were determined using a one-way ANOVA multiple comparisons test, and the significance is denoted by ^*^p < 0.05. Significant differences between saline and treated mice in **(B)** were determined using a one-way ANOVA multiple comparisons test, and the significance is denoted by ^**^p < 0.01 and ^***^p < 0.001.

To confirm the results of the initial study, B16-F10-LacZ-expressing tumor cells (2 × 10^5^) were injected intravenously (IV) in immunocompetent mice and treated (through IV) with a control peptide, the PD1 peptide antagonists (Peptide Combo), or anti-PD1 mAb (Figure 5A). The dose of peptides and α-PD1 mAb delivered was 200 μg/injection/mouse administered intraperitoneally (IP) on days 1, 2, 4, 6, 8, and 12 after the tumor injection. On day 14, lungs were harvested and the B16 nodules were counted. No adverse effects were observed within each treatment group. The PD1 peptide antagonists significantly reduced the number of B16 nodules from a mean of 81 in the saline-treated cohort to 28 (~65% inhibition). This effect was comparable with α-PD1 mAb, which showed a mean of 35 lung nodules (~57% inhibition) albeit not significantly different from the control peptide. Similar decreases in the number of nodules were seen when 5 × 10^5^ or 1.0 × 10^6^ B16 tumor cells were injected IV (data not shown).

Reduction in tumor metastasis after IV treatment of the PD1 peptide antagonists demonstrates potent efficacy. To identify whether similar efficacy may be attained using a lower dose and alternative route of administration, 2 × 10^5^ B16-F10 tumor cells were injected either IV or intradermally (ID) into mice (Figure 5B). Intradermal injection was chosen because drainage to the lymphatics is achieved via this route. The PD1 peptide antagonist dose for the IV group was 200 μg/injection/mouse, while the dose for the ID group was 20 μg/injection/mouse, which was one-tenth of the IV dose. In parallel with the initial B16-F10 study, peptides were administered on days 1, 2, 4, 6, 8, and 12 following tumor injection, with lungs harvested on day 14 and assessment of the number of B16 nodules. Confirming previous results (Figure 5A), IV administration of the PD1 peptide antagonists significantly reduced the number of B16 nodules from a mean of 36 in the saline-treated cohort to eight (~77% inhibition). Similarly, ID administration of the PD1 peptide antagonists at a fractional dose demonstrated a significant decrease in the number of B16 nodules, with a mean of 15 (~59% inhibition).

### PD1 Peptide Antagonists Alter Survival During Sepsis Correlating With Decreased Bacterial Burden and Improved Macrophage Function

**Figure 6:**
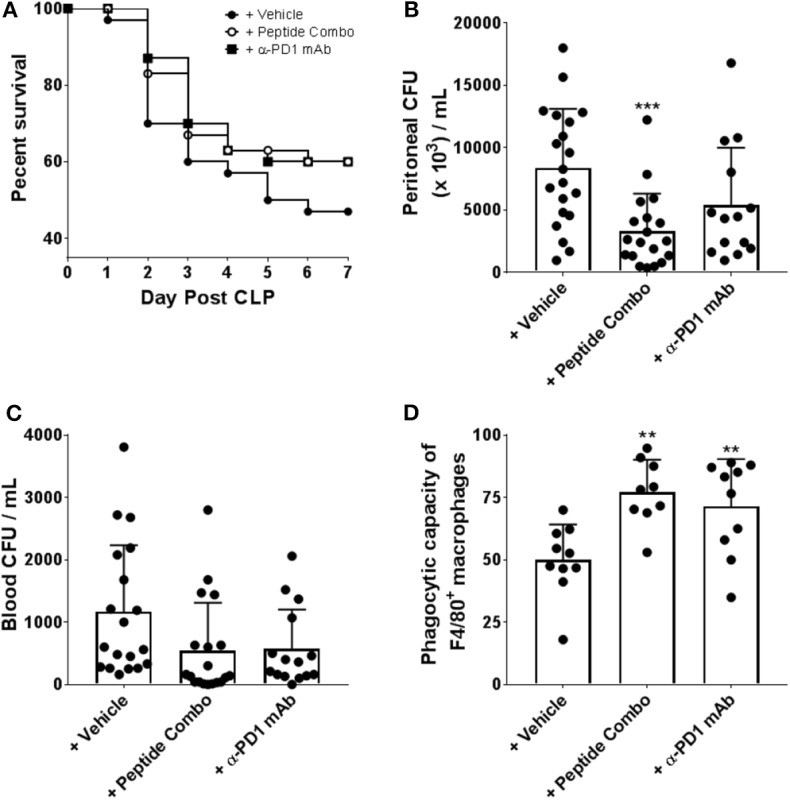
Enhanced survival, bacterial clearance and macrophage function in CLP induced septic mice following treatment with PD1 peptide antagonists. **(A)** PD1 peptide antagonist treatment enhances survival of CLP-induced sepsis in mice. Mice were subjected to CLP and treated with vehicle (*n* = 30), PD1 peptide antagonists (*n* = 30) or α-PD-1 mAb (*n* = 30) and survival was monitored for 7 days. The vehicle is a 0.1 M sodium acetate solution. Data were pooled from six independent experiments. **(B,C)** PD1 peptide antagonists treated mice had reduced bacterial burden after CLP. Bacteria levels were expressed as CFU per 1 mL. Data were pooled from five to seven independent studies (*n* = 20). Data are expressed as the mean ± SD. **(D)** Septic peritoneal macrophages from each group were fed fluorescently-conjugated *E. coli* and quantitative analysis of phagocytosis was measured by flow cytometry. Macrophages were identified as F4/80^+^. Data were pooled from four independent studies (*n* = 9–10). Data are expressed as the mean ± SD. Significant differences between saline and treated mice were determined using a one-way ANOVA multiple comparisons test, and are denoted by ^**^p < 0.01 and ^***^p < 0.001.

PD1-deficient mice have shown a role for PD1 in septic-induced immunosuppression (29). To determine whether the PD1 peptide antagonists alter experimental sepsis, C57BL/6 mice were subjected to cecal ligation and puncture (CLP) surgery and treated with PD1 peptide antagonists, and their survival was monitored. PD1 peptide antagonists were administered at a dose of 200 μg/injection/mouse IP twice daily on days 1, 2, and 3 following CLP. Anti-PD1 mAb was administered once daily on days 1, 2, 3, 4, and 5 following CLP at 200 μg/injection/mouse. In vehicle-treated mice, the survival rate was 47% (14 of 30 mice) at 7 days post-CLP (Figure 6A). However, the survival rate was slightly increased to 60% (18 of 30 mice) when mice were treated with PD1 peptide antagonists, which is similar to the survival rate in mice treated with α-PD1 mAb (Figure 6A).

In experimental sepsis, sustained/chronic infection contributes to disease by inducing excessive inflammation and immune dysfunction. To elucidate whether improved survival after the PD1 peptide antagonist treatment results from altered bacterial clearance, the levels of bacteria in the peritoneal cavity and in blood samples were measured. At 24 h post-CLP, vehicle-treated mice had significantly higher peritoneal cavity bacteria levels than mice treated with PD1 peptide antagonists (vehicle mean, 8381 colony forming units [CFU]/mL vs. PD1 peptide antagonists mean, 3316 CFU/mL; Figure 6B). Bacterial loads were also decreased with α-PD1 mAb treatment, but it was not significant and slightly higher than in the PD1 peptide antagonist group (Figure 6B). Comparison of blood bacterial loads between the groups demonstrated similar results with overall CFU counts in PD1 peptide antagonists and α-PD1 mAb-treated mice reduced (compared to the vehicle group), however, the differences were not significant (PD1 peptide antagonists mean, 540 CFU/mL; α-PD1 mAb mean, 576 CFU/mL; vehicle mean; 1168 CFU/mL; Figure 6C). Thus, treatment with the PD1 peptide antagonists showed an enhanced capacity to clear bacteria, at both a local and a systemic level.

Severe sepsis has also been shown to be closely associated with developing macrophage dysfunction, which is characterized by diminished bactericidal ability, decreased inflammatory cytokine production, and suppressed antigen-presenting function. To determine whether increased survival and reduced bacterial burden in PD1 peptide antagonist-treated mice was associated with relief from sepsis-induced dysfunction, peritoneal macrophages were isolated and their *ex vivo* phagocytic capacity was assessed in an *in vitro* assay system in which fluorescein-conjugated *E. coli* were incubated with the macrophages. PD1 peptide antagonist-treated mouse-derived macrophages demonstrated significantly greater phagocytic activity in comparison to that by vehicle-treated mouse-derived macrophages (PD1 peptide antagonists mean, 77% vs. vehicle mean, 50%; Figure 6D). Similarly, phagocytic activity of α-PD1 mAb-treated mouse-derived macrophages were significantly increased relative to the vehicle treatment (Figure 6D).

### PD1 Peptide Antagonists Enhance CD8^+^ T-Cell Immunogenicity and Protective Efficacy of a Rodent Malaria Vaccine

**Figure 7:**
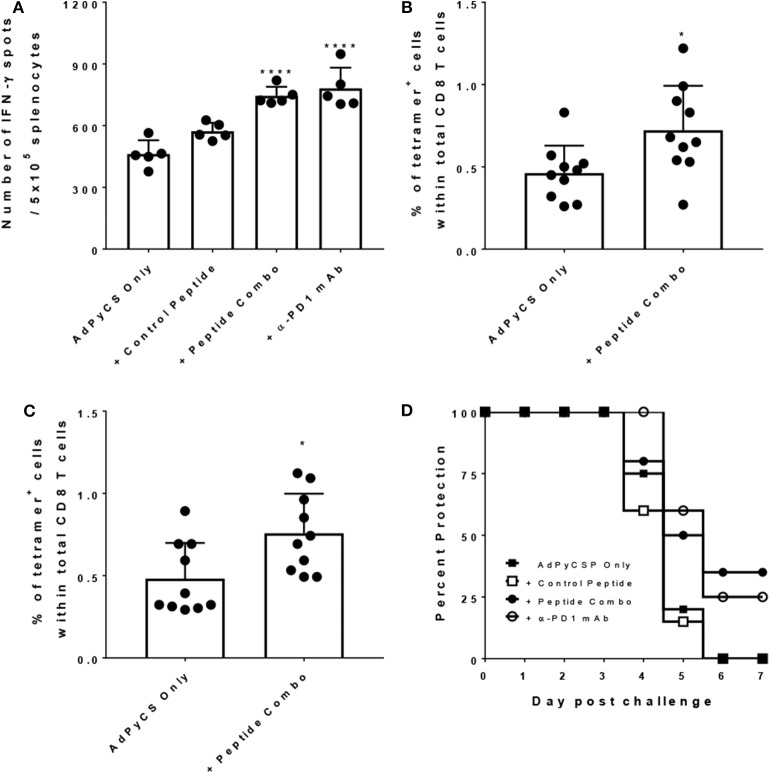
Increased CD8 T cell immunogenicity and efficacy following treatment with PD1 peptide antagonists. **(A)** At day 12 post-immunization, immunogenicity was assessed by measuring the number of splenic CS-specific, IFN-γ-secreting CD8 T cells using the ELISpot assay after stimulation with the H-2kd restricted CD8 epitope SYVPSAEQI. The control peptide is the OVA_323−339_ peptide. Data are expressed as the mean ± SD. Significant differences between AdPyCS alone and treated mice were determined using a one-way ANOVA multiple comparisons test, which are denoted by ^****^p < 0.0005. **(B)** On day 4 and **(C)** day 9, post-immunization splenocytes from mice were stained directly *ex vivo* for CD3, CD8, and SYVPSAEQI-specific tetramer and analyzed by flow cytometry. Data are expressed as the mean ± SD. Significant differences between groups were determined using unpaired two-tailed *t*-tests, which are denoted by ^*^p < 0.05. **(D)** Immunized and treated mice (*n* = 20/group) were challenged with Py 17XNL sporozoites IV. Data represent 2 independent experiments (*n* = 10/group) and are expressed as the mean percent protection. Parasitemia was assessed beginning on day 3 post-challenge and protection was defined as the absence of parasite detection in the blood by microscopic examination of Giemsa-stained thin smears.

To evaluate efficacy of the PD1 peptide antagonists as a prophylactic vaccine adjuvant, a rodent malaria model dependent on the generation of protective CD8 T cells was employed. T cell expansion and differentiation into effector and memory T cells is regulated by PD1, and this protein is rapidly upregulated upon naïve T-cell activation playing a regulatory role during naïve-to-effector T-cell differentiation (37). Additionally, in the context of vaccination, the extent of PD1 expression on activated T cells has been shown to be adjuvant-dependent (38). Thus, this data suggests that antagonizing PD1 during vaccination may increase immunogenicity and efficacy by limiting the natural downregulation that occurs when immune checkpoint inhibition is active. To evaluate whether the PD1 peptide antagonists alter antigen-specific CD8 T-cell responses during immunization, we used a well-established vaccine model of malaria (the recombinant replication-defective adenovirus expressing the entire *P. yoelii* circumsporozoite protein; AdPyCS), which is known to be protective (32). PyCS possesses an immunodominant H-2K^d^-restricted CD8^+^ T-cell epitope, SYVPSAEQI, in its C-terminal region. AdPyCS was injected intramuscularly (IM) into mice in the hindlimb without adjuvant, and the vaccinated mice were treated IP with a negative control peptide, the PD1 peptide antagonists, or α-PD1 mAb (Figure 7A). The dose of peptides and α-PD1 mAb delivered in this model was 200 μg/injection/mouse administered on days 1, 3, 5, and 7. On day 12, spleens were harvested and the number of splenic circumsporozoite protein (CS)-specific, IFN-γ-secreting CD8^+^ T cells were assessed using an ELISpot assay. The PD1 peptide antagonists significantly enhanced the number of CS-specific, IFN-γ-secreting CD8^+^ T cells relative to the AdPyCS immunization alone (Figure 7A). This increase was comparable to the α-PD1 mAb group for the demonstrated fold increase in the number of CS-specific, IFN-γ-secreting CD8^+^ T cells. To determine whether the increases in CS-specific, IFN-γ-secreting CD8^+^ T cells in response to PD1 peptide antagonist treatment were evident earlier in the response, the percentages of H-2K^d^/SYVPSAEQI-tetramer-specific CD8^+^ T cells were assessed at days 4 and 9 post-immunization (Figures 7B,C). At days 4 and 9 post-immunization, mice were treated twice (days 1 and 3) or four times (days 1, 3, 5, and 7), respectively, with PD1 peptide antagonists. As shown in Figures 7B,C, PD1 peptide antagonists significantly enhanced the percentage of splenic tetramer^+^ CD8^+^ T cells relative to AdPyCS immunization alone at both days 4 and 9 post-immunization. These data demonstrate that antagonizing PD1 using a prophylactic vaccination enhances the expansion of antigen-specific T cells. To elucidate whether the increased immunogenicity to AdPyCS is associated with enhanced protection, challenge studies were conducted in the presence of PD1 antagonists. Mice were immunized IM with a suboptimal dose (10^9^ virus particles) of AdPyCS, which does not result in protection. At days 1, 3, 5, and 7 post-immunization, mice were treated IP with 200 μg of negative control peptide, the PD1 peptide antagonists, or α-PD1 mAb. Twelve days post-immunization, mice were challenged IV with *P. yoelii* sporozoites. Parasitemia was assessed via blood smears beginning on day 3 post-challenge. As shown in Figure 7D, no protection was measured in AdPyCS alone, or in mice treated with a negative control peptide. However, the PD1 peptide antagonist treatment resulted in 35% protection. Protection was also increased with α-PD1 mAb treatment, but it was slightly lower than the PD1 peptide antagonist group (Figure 7D).

## Discussion

Our goal was to identify novel peptide scaffolds that are able to antagonize the PD1 receptor to interrogate the utility of the checkpoint receptor inhibitors as a T-cell adjuvant that can enhance the immunogenicity and protective efficacy of certain infectious disease vaccines by expanding vaccine-induced CD8^+^ T-cell responses. As the dominant checkpoint drug modality, mAbs have several practical drawbacks when combined with vaccine formulations in some disease indications. Most important, are the safety concerns related to severe immune-related adverse events, which are likely a result of the sustained PD1 inhibition because of a half-life of 15–20 days and >70% receptor occupancy for months (24, 25, 39–41). While these types of adverse outcomes are often tolerable in an oncology setting, the risk–benefit ratio of potentially trading an autoimmune disease from a checkpoint mAb for the opportunity to protect against infectious diseases domestically and in the developing world is high, especially considering the risk to childhood vaccination strategies (42). In our current studies, we identified four 20-mer peptides that were bound to the human PD1 receptor by screening a phage display library and subjecting them to further characterization. Binding and biological activity of the peptides was confirmed by their ability to block the binding of PD-L1 in a Jurkat T cells reporter assay and to enhance T-cell effector function in a tetanus toxin antigen recall assay from vaccinated human PBMCs.

Confident that the peptides specifically bound to PD1 and had antagonistic activity, we obtained some structural and analytical clues about the peptides that were bound to the PD1 receptor using *in silico* modeling. We predicted that each peptide was bound to a unique position on the receptor with some overlap and that this was consistent with the measured peptide activities. This structural information has provided valuable clues about the functional site of the receptor and the structural relationship to other members of the B7 and CD28 super families of receptors (e.g., PD1, CTLA4, ICOS), which can be used to further fine-tune these peptide scaffolds (e.g., SAR); this subject is motivating future studies. Additionally, the KD value for the interaction of the peptides to the human PD1 receptor was found to be in the range of 10^−4^-10^−5^ compared to micromolar KD values reported for PD-L1 interactions with human PD1 (43). The lower affinity of the peptides compared to the natural ligand and their non-overlapping interaction sites on PD1 prompted us to test the combination of the four peptides *in vivo*. Thus, we showed that their bioactivity was equivalent to an anti-PD1 mAb in the B16-F10 syngeneic mouse melanoma model. While the standard IV route worked well for our peptides in this model, we demonstrated that we could provide a much smaller dose via intradermal administration, which is a more useful route for infectious disease vaccine delivery.

Sepsis represents an intriguing system for the modeling of PD1 antagonistic therapeutic activity. Sepsis is a syndrome that is triggered by pathogen factors, but that is driven by the dysregulation of the host immune response (44). Immunosuppression, which is also known as “sepsis-induced immunoparalysis,” appears to be the lethal component of sepsis, rather than excessive immune activation (45) in which immunotherapies have shown preclinical and clinical promise with anti-PD1 mAbs having an effect in small clinical trials (46). Our peptides improved overall survival and decreased bacterial burdens in mice, which correlated with rescued macrophage activity. Infectious diseases such as non-typhoid salmonella, pneumococcal infections, HIV, malaria, and dengue are associated with an increased risk of sepsis and septicemia, and most mortality in children under 5 years is attributable to sepsis (46–50). Therefore, therapeutic application of PD1 checkpoint inhibitors in septic animals provides valuable insight for chronic or seasonally infectious diseases and their ability to restore exhausted T-cell function. The function of the checkpoint inhibitors to reverse exhaustion and to allow one's own immune system to fight infection is central to the immunotherapy revolution in cancer, in which the immune system is recruited to recognize and attack cancer cells rather than directly trying to kill the cancer cells through chemical agents or radiation. This suggests that infectious agents are actively impeding the host's immune defenses (for example, cancer cells), however, a direct mechanism of immune evasion remains unknown.

**Figure 8:**
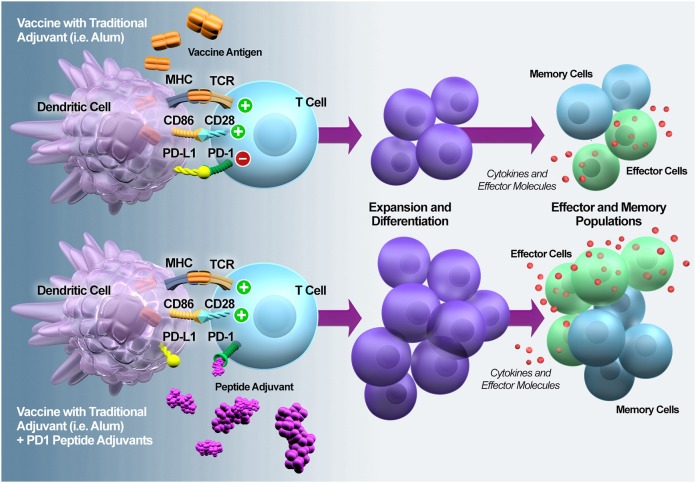
PD1 antagonization potentiates T cell expansion and differentiation into effector and memory T cells. PD1 is rapidly upregulated upon naïve T cell activation and it plays a regulatory role during naïve-to-effector T cell differentiation (37). In the context of vaccination, MacLeod et al. (38) demonstrated that the magnitude of PD1 expression on the activated T cells is adjuvant-dependent. Thus, adding a PD1 peptide adjuvant during the early phase of vaccine-induced T cell activation should modulate expansion and differentiation, resulting in enhanced numbers and functionality of effector and memory T cells.

Along with alleviating immune exhaustion that results from infection, checkpoint inhibitors have also been suggested to be ideal T-cell adjuvants because they have been shown to drive the differentiation of antigen-specific effector and memory T cells (Figure 8). We showed that the addition of the peptide-based PD1 antagonists to one of the established malaria vaccination regimens ostensibly expanded and doubled the level of malaria-specific CD8^+^ T-cell response and improved the survival of mice upon malaria challenge. While we showed the efficacy of our peptides as a T-cell adjuvant, it is important to also note that a prophylactic vaccine to a disease such as malaria in holoendemic regions will likely have to contend with ongoing infections. Therefore, the peptide-based PD1 inhibitors would not just provide an adjuvant effect for the T-cell; it would also alleviate the existing T-cell exhaustion that results from an ongoing heavy load of infection and allow the T cells to be responsive to the vaccine antigens.

Many pathogens have been remarkably responsive to the very basic concept of training the immune system to recognize the pathogens when they re-encounter them, which follows the natural course of infection. Even more confounding are the pathogens that resist any type of vaccination, including sterilizing immunity to natural infection (e.g., malaria) (51). Strikingly, there is no highly effective prophylactic vaccine against parasitic pathogens, despite decades of investigation and investment. Like tumors, these pathogens are tolerated by our immune system, which can lead to deadly consequences for the hosts. For example, in areas of Africa where people are infected with malaria continuously or seasonally, individuals can become tolerant to the parasite infection (52). However, if an individual spends several seasons without contracting the parasite, the immune system reverts back to a naïve state (53, 54), strongly suggesting that parasites are actively modulating our immune memory-building capacity, which has yet to be identified. Therefore, it is our hypothesis that the checkpoint receptors may be a direct target for counteracting a parasite's immune evasion tactics. Thus, by combining inhibitors of the checkpoint pathway as an adjuvant with antigens specific to the parasite, we may allow direct expansion of antigen-specific memory and effector CD8^+^ T cells, which are absent in current vaccine adjuvants that generally focus on enhancing Th2 responses.

Modern vaccinology has been working to replace whole cell/virus vaccines with more reductionist (i.e., split, subunit, and recombinant) approaches because of issues raised with reactogenicity resulting from the presence of lipopolysaccharides and nucleic acids. As the safety profiles in modern vaccines has improved, immunogenicity has decreased, which is exemplified by acellular pertussis and influenza vaccines (55). Additionally, subunit vaccines skew the response to a primarily Th2 response at the expense of Th1 and near absence of a CD8^+^ T-cell engagement. Unfortunately, while adjuvants are generally designed to trigger innate inflammatory danger signals, resulting in local and systemic reactogenicity (i.e., saponins, oil emulsions, and TLRs), they lack the capacity to mimic the immune signature of the natural infection (56, 57), likely because multiple danger signals are required. Thus, it is of utmost importance that our PD1 peptides act as a potent CD8^+^ T-cell adjuvant for vaccines against pathogens. Unlike traditional adjuvants, which primarily trigger an innate and non-specific immune response to the vaccine antigen, inhibiting checkpoint receptors may trigger a direct expansion of the CD8^+^ T-cell compartment that is specific to administered antigens. Unlike the anti-PD1 mAb therapies, our PD1 peptides are more labile *in vivo* (data not shown), which fits within an immunological window of antigen presentation and T-cell expansion but does not remain for weeks to cause autoimmune events. Finally, because these are linear peptides, it is another notable advantage that we can easily encode them into nucleic acid vectors to control the duration of their release and to decrease the cost, which will be necessary for indications such as malaria and other tropical diseases.

## Data Availability Statement

The raw data supporting the conclusions of this article, except for proprietary peptide sequences, will be made available by the authors, without undue reservation, to any qualified researcher.

## Ethics Statement

All procedures were performed in accordance with strict institutional guidelines for animal care and use. Protocols were conducted in accordance with National Institutes of Health guidelines and with approvals from the Institutional Animal Care and Use Committee of Rhode Island Hospital (#5025-17) and from the Institutional Animal Care and Use Committee of Rockefeller University (Assurance no. A3081-01). A human PBMC sample was obtained from a donor through an informed consent protocol approved by the Western Institutional Review Board.

## Author Contributions

Conceptualization and study design were contributed by GG, VK, TP, JP, and MT. Data organization and formal analyses were performed by GG, VK, TP, CB, and MM. Methodology and experiment executions were performed by CR, AM, GW, RR, MS, AA, JH, and MT. Supervision was contributed by GG, VK, TP, JP, and AN. Writing the original draft was completed by GG, VK, TP, CB, and MT. Manuscript review and editing was performed by JP, MM, AN, KT, JC, CR, AM, RR, MS, C-SC, and AA.

### Conflict of Interest

VK, TP, CB, JP, AN, KT, JC, and GG were employed by the company Leidos, Inc. MM was employed by the company MM Scientific Consultants, Inc. CR was employed by Inovio Pharmaceuticals. AM, GW, RR, and MS were employed by IMV, Inc. The remaining authors declare that the research was conducted in the absence of any commercial or financial relationships that could be construed as a potential conflict of interest.
