# Supplementary material for: Novel Peptide-Based PD1 Immunomodulators Demonstrate Efficacy in Infectious Disease Vaccines and Therapeutics
Source: Front Immunol. 2020 Mar 6;11:264. doi: 10.3389/fimmu.2020.00264 (PMC7068811; doi:10.3389/fimmu.2020.00264)
Supplement: Supplementary file 1 [file Data_Sheet_1.docx]

**Supporting Information**

**S1 Fig.**

**Phage Enrichment**

| Round | Conditions | Input | Output | Enrichment Factor |
| --- | --- | --- | --- | --- |
| 1st | Target protein: 30 ug/ml PD1  Washing: 0.1% Tween-20 TBST, 10 times  Elution: Glycine-HCl + BSA pH 2.2  Pre-counterselection: 2%Milk-TBS | 3.0 × 10^11^ | 1.2 × 10^4^ | 2.5 × 10^7^ |
| 2^nd^-P | Target protein: 30 ug/ml PD1  Washing: 0.1% Tween-20 TBST, 10 times  Elution: Glycine-HCl + BSA pH 2.2  Pre-counterselection: 2%Milk-TBS | 5.3 × 10^11^ | 1.1 × 10^4^ | 4.8 × 10^7^ |
| 2^nd^-N | Target protein: No coating  Washing: 0.1% Tween-20 PBST, 10 times  Elution: Glycine-HCl + BSA pH 2.2  Pre-counterselection: 2%Milk-TBS | 6.7 × 10^10^ | 3.9 × 10^2^ | 1.7 × 10^8^ |
| 3^rd^-P | Target protein: 30 ug/ml PD1  Washing: 0.1% Tween-20 PBST, 10 times  Elution: Glycine-HCl + BSA pH 2.2  Pre-counterselection: 2%Milk-TBS | 4.3 × 10^10^ | 3.5 × 10^4^ | 1.2 × 10^6^ |
| 3^rd^-N | Target protein: No coating  Washing: 0.1% Tween-20 PBST, 10 times  Elution: Glycine-HCl + BSA pH 2.2  Pre-counterselection: 2%Milk-TBS | 5.3 × 10^9^ | 5.6 × 10^2^ | 9.5 × 10^6^ |
| 4^th^-P | Target protein: 30 ug/ml PD1  Washing: 0.1% Tween-20 PBST, 10 times  Elution: Glycine-HCl + BSA pH 2.2  Pre-counterselection: 2%Milk-TBS | 2.4 × 10^11^ | 3.2 × 10^5^ | 7.6 × 10^5^ |
| 4^th^-N | Target protein: No coating  Washing: 0.1% Tween-20 PBST, 10 times  Elution: Glycine-HCl + BSA pH 2.2  Pre-counterselection: 2%Milk-TBS | 3.0 × 10^10^ | 3.3 × 10^3^ | 9.0 × 10^6^ |
| 5^th^-P | Target protein: 30 ug/ml PD1  Washing: 0.2% Tween-20 PBST, 10 times  Elution: Glycine-HCl + BSA pH 2.2  Pre-counterselection: 2%Milk-TBS | 3.2 × 10^11^ | 7.2 × 10^6^ | 4.4 × 10^4^ |
| 5^th^-N | Target protein: No coating  Washing: 0.2% Tween-20 PBST, 10 times  Elution: Glycine-HCl + BSA pH 2.2  Pre-counterselection: 2%Milk-TBS | 4.0 × 10^10^ | 9.9 × 10^4^ | 4.0 × 10^5^ |

**S1 Fig. Phage Enrichment summary table for all five panning rounds.** The enrichment factor is the ratio of the input to the output phage (phage forming unit). The enrichment factors were monitored over the course of 5 panning and serve as relative indicators that PD1-binding phages are being selected. N = not coated with PD1 and P = coated with PD1.

**
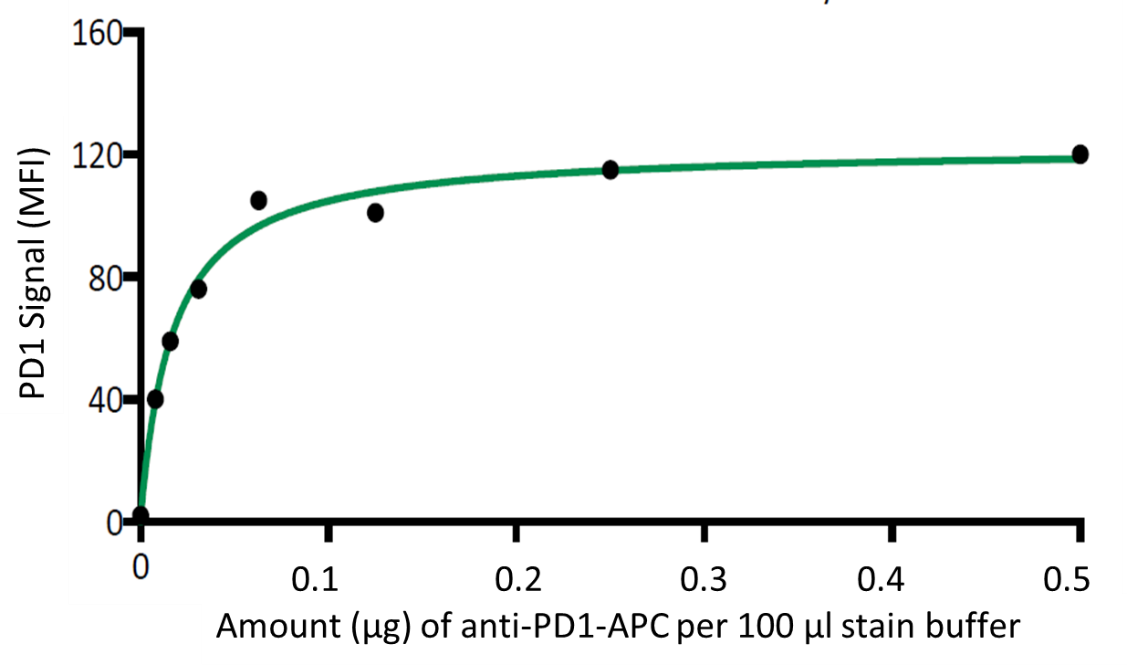
**

**S2 Fig. Anti-PD1 antibody binding to Jurkat cells that overexpress human PD1.** Different concentrations of anti-PD1 antibody conjugated to APC were incubated with Jurkat cells that overexpress human PD1. Mean fluorescence intensities were measured by flow cytometry.


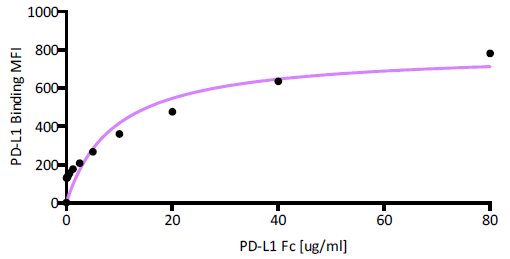


**S3 Fig. PD-L1 Fc binding to PD1-overexpressing Jurkat cells in a dose-dependent manner.** Different concentrations of PD-L1-Fc fusion protein were incubated with Jurkat cells that overexpress PD1. Binding of PD-L1-Fc was detected with anti-human Fc antibody conjugated to AF647. Mean fluorescence intensities were measured by flow cytometry. A sub-saturating level of 4 µg/mL of PD-L1 Fc fusion protein was selected for use in the competition experiment with PD1 peptides.


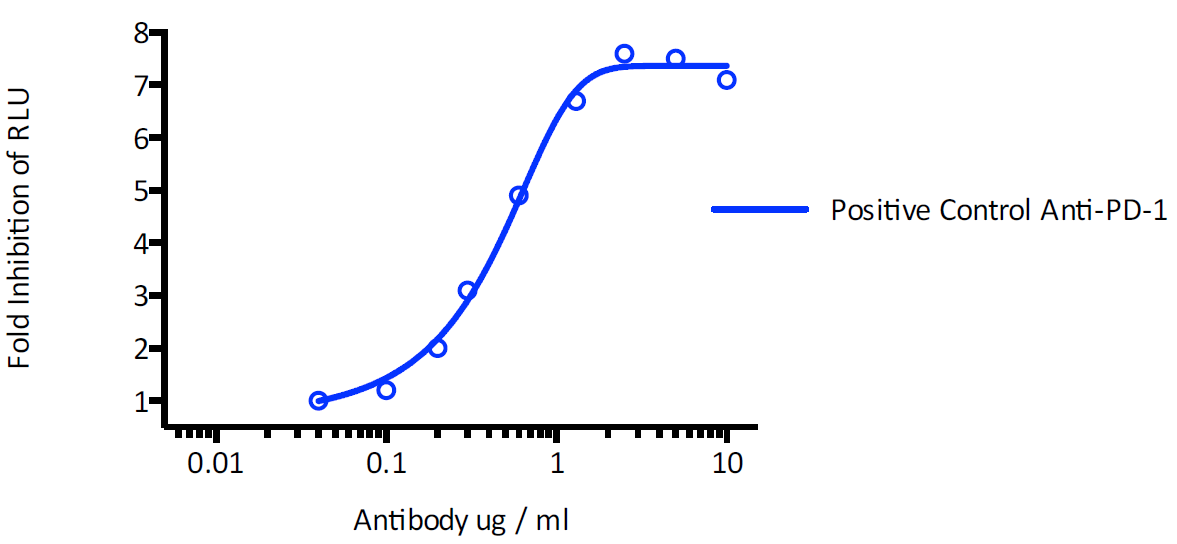


**S4 Fig. Anti-PD1 neutralizing antibody inhibits the interaction of PD-L1 (expressed on CHO cells) with PD1 (expressed on Jurkat cells) in a dose-dependent manner.** Luminescence signals were measured and normalized to the signals observed in cells not treated with antibody (fold inhibition).
